# Making Come-Alive and Keeping Un-Alive: How People Relate to Self-Guided Web-Based Health Interventions

**DOI:** 10.1177/1049732320902456

**Published:** 2020-02-11

**Authors:** Marianne T. S. Holter, Ottar Ness, Ayna B. Johansen, Håvar Brendryen

**Affiliations:** 1 The Norwegian Centre for Addiction Research (SERAF), Faculty of Medicine, University of Oslo, Oslo, Norway; 2 Department of Education and Lifelong Learning, Norwegian University of Science and Technology, Trondheim, Norway

**Keywords:** eHealth, mHealth, web-based interventions, working alliance, therapeutic alliance, qualitative, grounded theory, interviewing, Europe, Scandinavia, Norway

## Abstract

Health interventions delivered online (self-guided web-based interventions) may become more helpful through a person-to-program “working alliance.” In psychotherapy, the working alliance signifies a therapeutically useful client–therapist relationship and includes an emotional bond. However, there exist no theories of how program users relate to online programs, or that explain a person-to-program bond theoretically. Addressing this gap, we conducted qualitative interviews with and collected program data from users of a self-guided web-based intervention. Taking a grounded theory approach, the analysis arrived at a model of relating based on two relational modes—*making come-alive* and *keeping un-alive*. Different combinations of these modes could describe a range of ways of relating to the program, including a *nonsocial interaction*, a *semi-social interaction*, and a *semi-social relationship.* A person-to-program bond is explained by the model as an *experienced supportive social presence*, enabled by making come-alive and a positive program interaction.

Online health interventions may dramatically change how health care is provided by providing high-quality, individualized care without the expensive use of human resources, available to anyone with the access to the internet, anytime and everywhere. Often called “eHealth” or “mHealth” programs, these computerized interventions can be completely automated and have repeatedly proven to be effective (Barak et al., 2008; Deady et al., 2017; Knowles & Mikocka-Walus, 2014; Shahab & McEwen, 2009). However, self-guided web-based interventions are sometimes referred to as “black boxes,” because surprisingly little is known of how they work (Danaher et al., 2015; Michie & Abraham, 2008; Webb et al., 2010). To harness the full potential of these programs, there is a need to explore their working mechanisms. Intriguingly, one proposed working mechanism is a person-to-program “working alliance,” or collaborative relationship. This proposition is derived from psychotherapy, where the therapeutic value of a working alliance is widely acknowledged (Barber et al., 2009; Doran, 2016; Flückiger et al., 2018; Horvath & Luborsky, 1993; Wampold, 2015). In the case of self-guided web-based interventions, the role of the therapist is assumed by the computer program, and the possibility of a person-to-program alliance has intrigued researchers in the field (Barazzone et al., 2012; Baumel et al., 2017; Clarke et al., 2016; Ormrod et al., 2010; Peck, 2010).

There is evidence suggesting the existence of a person-to-program alliance (Bickmore, Gruber, & Picard, 2005; Kiluk et al., 2014; Meyer et al., 2015). For example, Bickmore, Gruber, and Picard (2005) were able to influence participants’ alliance to a self-guided web-based intervention through changing intervention design. The program’s main feature was an embodied relational agent, “Laura,” and alliance toward the program was stronger for participants who received a version in which Laura performed “socio-emotional behaviors” than for participants who received a version that was purely goal-oriented. In another study, Meyer et al. (2015) found an association between alliance to a self-guided web-based intervention and outcome: Depression scores were lower for participants with a strong alliance to the program, even when controlled for early symptom reduction.

However, there is no consensus of exactly what a person-to-program alliance is—what elements it includes. In psychotherapy, the alliance is often defined as comprising three elements: (a) that client and therapist agree on the goals of therapy (e.g., changing a specific behavioral pattern vs. resolving childhood conflicts); (b) agreement on the therapeutic tasks toward that goal (e.g., social skills training vs. free association); and (c) the emotional bond, or “the nature of the human relationship between therapist and patient” (Bordin, 1979, p. 254). An emotional bond has been described as feeling understood, cared for, appreciated, and comfortable with, as well as involving respect, honesty, liking, trust, and attachment (Bordin, 1979; Horvath & Greenberg, 1989). Thus, the experience of an emotional bond appears to imply another social actor with thoughts and feelings, and therefore seems an unlikely element of an alliance to an inanimate computer program. A computer program, after all, is no human being.

On a behavioral level, people do seem to respond to computers as if they were other humans in a variety of situations, as demonstrated in a series of experiments conducted by Nass and Reeves (1996). Their overall finding was that participants interacted with computers in accordance with social norms for human interaction (e.g., being polite to computers or disliking computers that criticized other computers). Nass and Reeves call this the “media equation”—media equals real life—and explain it by observing that “people are not evolved to twentieth-century technology,” and therefore automatically behave socially toward anything that acts socially toward them (heading: “Why do people respond naturally and socially to media?”—third paragraph).

On a more cognitive and emotional level, there are vivid examples in the literature of program users sometimes thinking of self-guided web-based interventions as other human beings (Bickmore, Caruso, et al., 2005; Bickmore, Gruber, & Picard, 2005; Brandt et al., 2013; Kaplan et al., 2003). Two of these studies are especially relevant for the current article: In one qualitative study, Brandt et al. (2013) interviewed participants (*N* = 9) who had used a self-guided web-based intervention for quitting smoking. The intervention mainly consisted of an interactive, tailored website on which the participant could navigate through different pages. Nevertheless, the authors found that participants could “project human qualities” to the program and interact with it as if it were a person. Some of the data excerpts clearly demonstrate this (e.g., “you grow a relationship to the program” and “I miss the care even though I know it’s just a machine”). In a different study, Kaplan et al. (2003) interviewed participants (*N* = 14) who used a self-guided web-based system. The system was placed in their homes, served a variety of health purposes, and participants could call the intervention, upon which the intervention answered with a human voice (recorded by an actor). Participants could respond by pressing numbers on their phone. The authors found that participants formed three types of relationship to the web-based intervention: “feelings of love” (*N* = 3), described as talking about the program as a person (a “friend,” “helper,” or “family member”); “feelings of guilt” (*N* = 9), described as feeling judged or lectured by the program; and “ambiguity and ambivalence.” In this latter category, six participants talked about the intervention as a machine, one participant talked about the intervention as “he” instead of “it,” and four participants expressed ambiguity as to whether they were interacting with a person or a program.

The evidence that these studies report may be the experiences of a few unique individuals. Furthermore, Kaplan et al.’s (2003) category “feelings of love” seems to suggest a stronger and more complex emotion than the authors provide evidence for in the excerpts. This potential overstatement raises questions of whether there may have been nuances to the participants’ emotional experiences with the intervention that are lost in the analysis. Finally, because these studies’ aims were not to conceptualize the emotional bond of a potential person-to-program alliance, they do not go into depth in explaining how or why the documented experiences might arise, nor how they compare with the traditional conceptualization of an “emotional bond.”

In sum, there is a need to improve our understanding of the working mechanisms of self-guided web-based interventions (Danaher et al., 2015; Michie & Abraham, 2008; Webb et al., 2010), and one possible working mechanism is a person-to-program working alliance, which theoretically should involve an emotional bond (Bordin, 1979). Indications of a person-to-program emotional bond include one study’s successful manipulation of this bond through program design (Bickmore, Gruber, & Picard, 2005) as well as case evidence of people thinking of such computer programs as people (Bickmore, Caruso, et al., 2005; Bickmore, Gruber, & Picard, 2005; Brandt et al., 2013; Kaplan et al., 2003). However, it is uncertain whether these documented examples are a collection of a few, extreme cases, or whether they represent a more common psychological process. Furthermore, the seemingly paradox of experiencing an emotional bond to an inanimate web-based intervention must be explored and theoretically explained. Thus, the purpose of the current study was to develop a theoretical model of how people relate to self-guided web-based health interventions and to discuss the implications of this model for a potential person-to-program emotional bond. In pursuing this purpose, we conducted a grounded theory study (Charmaz, 2014) with the users of a self-guided web-based intervention for quitting smoking, pursuing the following research question: *How do the users relate to the intervention?*

## Method

### Qualitative Approach and Epistemological Stance

We chose grounded theory (Charmaz, 2014) because we wanted to focus on the participants’ ways of relating to the self-guided web-based intervention as a process. Relating can be viewed as a creative process in which the individual creates his or her interpretation and experience of the relationship (Winnicott, 1969). Instead of describing this act of creating in terms of its product (i.e., the experience of relating), we wanted to focus on the creative acts themselves. Targeting processes made grounded theory a suitable approach, as did the method of constant comparison to keep the analysis close to the data (Charmaz, 2014; Creswell, 2013). Furthermore, because relational processes in automated therapy is an underresearched area, we considered it most appropriate to retain a flexible approach to analysis, making the dynamic and emerging approach advocated by Charmaz (2014) a suitable choice.

The study was conducted within a critical realist perspective (Maxwell, 2013). That is, in our pursuit of a relational model for automated therapy, we assumed that it is possible to understand how people actually relate to a self-guided web-based intervention (realist ontology), instead of only attending to how participants constructed “relating” through language, norms, and roles (constructivist ontology). However, the stance of critical realism differs from “pure” realism in that it acknowledges the interpretative stance of the knowledge producers, and that all efforts to describe reality will always, at best, be partial (constructivist epistemology). In other words, we believe the reality of how people experience and relate to self-guided web-based interventions can be described and explained, and that any model that attempts to do so will be one of several possible representations of their real experiences. This implies that the model we propose can be discussed as a more or less valid explanation of the participants’ experiences. We will return to this in the discussion of the study’s validity.

### The Self-Guided Web-Based Intervention: “Andy” (“Endre”)

**Figure 1.**
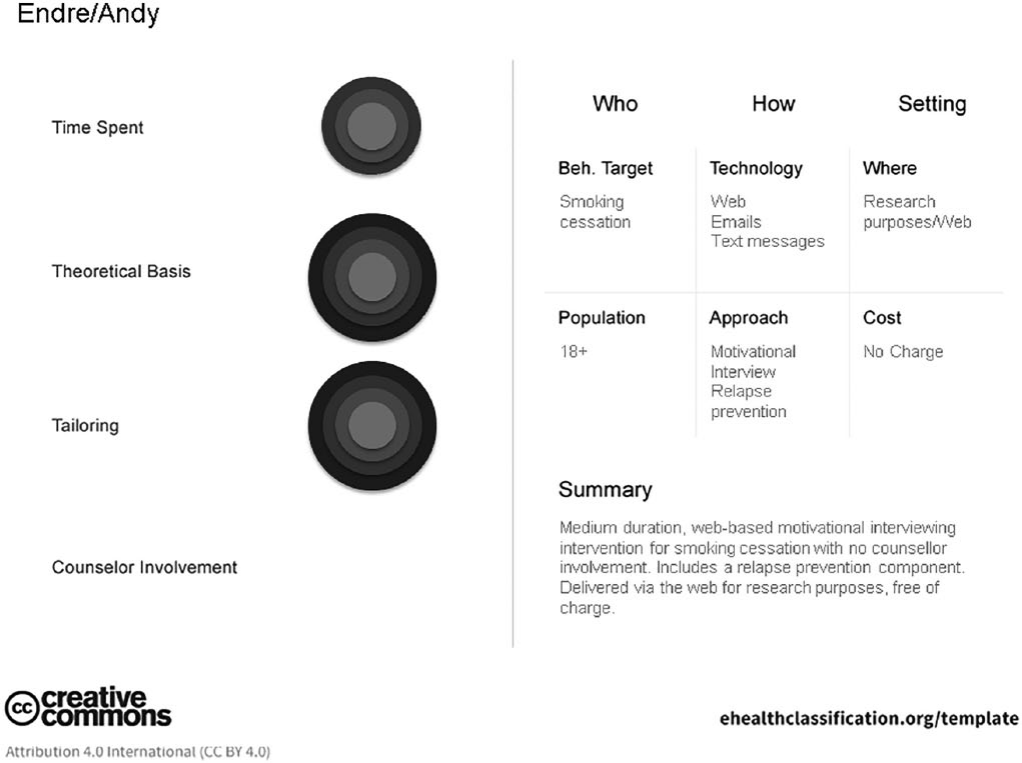
The self-guided web-based intervention “Andy” as described with the eHealth-classification tool of Bewick and colleagues (2017).

“Endre” (“Andy”) is a Norwegian, self-guided web-based intervention for quitting smoking designed to support a working alliance (Holter, Johansen, & Brendryen, 2016). Some important features of the intervention are summarized in Figure 1 (Bewick et al., 2017). It is primarily web-based; the users receive emails with links that log them onto the program online. The intervention’s name, “Endre,” is a Norwegian masculine name that also means “to change.” The name was chosen to support an experience of the program as a relational agent (Bickmore, Puskar, et al., 2010), and was thus one alliance-supporting design feature. To better communicate this relational agent to non-Norwegian readers, the intervention will be called “Andy” in this article. Andy is a nonembodied relational agent; that is, the program is text-based, and Andy is never visualized through any pictures, videos, or drawings. Instead, Andy “writes” in the first-person tense, referring to “himself” as “I,” and engages the program user in a written “conversation.” This “conversation” is dynamically tailored to the user’s input. Other than the relational agent, alliance-supporting elements include “alliance factors” (Barazzone et al., 2012; Cahill et al., 2008) that are embedded into the program (e.g., communicating with warmth, empathy, and unconditional acceptance, and providing feedback to the user’s input) and Andy’s use of (computerized) Motivational Interviewing (Miller & Rollnick, 2012). A detailed description of the intervention’s rationale, including how it is intended to support an alliance, can be found in Holter et al. (2016).

Andy consists of a phase of preparing for quitting smoking and a follow-up phase after the user has quit. In the standard version, the preparation phase consists of 10 different session over 10 days, followed by a follow-up phase of 14 different sessions over 4 weeks. The actual number of sessions each program user receives is tailored to individual use, as the program adapts to how often the user logs on. Once the user has quit, she or he enters a follow-up phase in which she or he in addition to web-based sessions also receives text messages from Andy in the evening, asking whether she or he has been smoke-free that day. If the user reports a lapse, she or he is given access to a special session aimed at preventing the lapse from becoming a full-blown relapse to smoking. A typical session consists of Andy greeting the user, introducing today’s quitting-related topic, and asking a topic-related question, which the user must answer by choosing a multiple-choice alternative. Sometimes the user is asked to write his or her answer in a textbox. Some of these user-generated texts are presented to the user at a later point, in a different “conversation” in which Andy “remembers” something the user has said previously. Next, Andy will respond to the user’s answer, and the “conversation” will go back and forth, ending by Andy summarizing today’s session and bidding farewell.

### Participants and Recruitment

The study was conducted in Norway and is based on two different types of data from two different subsamples: an interview sample and a noninterview (“reflection note”) sample. For both samples, we recruited people who wanted to quit smoking with the help of a web-based intervention. In the following, we will describe the two samples separately.

The interview sample (*N* = 16) was recruited via the researchers’ social network on Facebook, a popular discussion forum in Norway (“Underskog”), and a local general practitioner’s (GP) office. This convenience sample of 16 participants was fairly heterogeneous, including both men (*N* = 5) and women (*N* = 11), varying in age (32–70 years), and holding different occupations (including nurses, construction workers, and people in higher education). Some participants had stopped using the program while still preparing for quitting, some completed the program, some had successfully quit smoking, some had not, and yet others had relapsed. Thus, this study sample was diverse on several parameters, and the ensuing model is based on and includes these different perspectives. Three of the participants were distant acquaintances of the interviewer (Marianne T. S. Holter); this did not seem to affect the data negatively.

The noninterview sample (*N* = 55) participated through “reflection notes,” a special type of written activity in the program described below. These participants were originally recruited for a different, quantitative study, but they consented to the current use of their program data. They were recruited through Healthy life centers in several Norwegian municipalities as well as through advertisements on Facebook, Google, and online newspapers. The first 112 participants who consented were included in a sample pool for the current study, from which we theoretically sampled (Charmaz, 2014) 55 participants; this process is detailed in the “Findings” section. The reflection note sample was also diverse, including men (*N* = 10) and women (*N* = 45) of different ages (25–66 years) and with different levels of education (ranging from primary school to a higher university degree of 4 years or more).

### Data Collection

#### Interviews

Of the 16 participants who were interviewed, three were interviewed twice to elaborate on questions that arose in the initial analysis of their first interview. The interviews were semi-structured and lasted between 35 and 80 minutes. Eight participants were interviewed face-to-face in various settings (at the interviewer’s office, at the participant’s home, at the participant’s workplace, and at cafés). The other eight participants were interviewed over the telephone, as they lived in other parts of the country. All interviews except three were audiotaped; in these interviews, a recording device was for various reasons not available, and the interviewer instead made notes, careful to separate quotes from notes.

After the first six interviews, we made considerable changes to the interview guide. These first interviews served two purposes: data collection for the current study and gathering feedback for program improvement. However, we experienced substantial challenges in getting rich data on how the participants related to the program, leading us to revise the interview guide. In the revised interview guide, the participants were first asked to give a free account of their experiences. Next followed a section where the participants and interviewer together reflected on questions of relating to Andy, both active in the discussion from their unique vantage points, ultimately seeking general knowledge about the processes under study (“epistemic interviewing”; Brinkmann, 2007). This section also included interview vignettes (Barter & Renold, 1999; Finch, 1987; Jenkins et al., 2010) that exemplified four different ways of relating to Andy (based on the preliminary analysis), as well as other interview techniques. We discuss several of these techniques in a separate publication on how qualitative interviews can be used to explore potential working mechanisms in self-guided web-based interventions (Holter, Johansen, et al., 2019).

#### Reflection notes

In addition to changing the interview guide, the challenges we faced in getting rich data caused us to include a second data source which we call “reflection notes.” Reflection notes were collected within the program sessions as a response to selected questions: At the end of three different sessions, Andy asked the participants, “How would you describe working with me?” and at the end of the intervention, Andy asked for feedback. Participants answered each question in a text box (usually with one to four sentences). It is this written material that is referred to as “reflection notes.”

Reflection notes were included in the later stages of the study to increase its validity through methodological triangulation (Maxwell, 2013), and to saturate the analysis (Charmaz, 2014). One reason for including reflection notes was that early analysis indicated that participants might find it difficult to talk about how they related to Andy in the social interaction of the interview. We reasoned that this embarrassment might influence what the participants were willing to share in the interview, threatening the validity of the interview data. By gathering reflection notes from within the program activity itself, we were able to exclude the social situation of the interview from this data material, making reflection notes a useful complementary data source to the interviews. The categories and patterns developed from the interview data were found in the reflection note data as well, strengthening our confidence in the interview data’s validity.

### Data Analysis

We used grounded theory (Charmaz, 2014) to analyze the data. Audio recordings were transcribed on what was deemed a necessary level of detail to answer the research questions (Bailey, 2008; Bird, 2005). Because the interviewer also transcribed, unclear speech or ambiguous statements could be complemented by her memory of the encounter and postinterview memos (Bird, 2005). The interview transcripts were coded inductively using HyperResearch. Codes were then sorted and organized into higher-order codes through mind-maps, tables, and memos (Charmaz, 2014; Miles et al., 2014). In this early inductive phase, we also made brief case analyses of participants. These case analyses were 1- to 2-page documents that summarized the case and provided tentative answers to the research questions for each participant (Miles et al., 2014). Case analyses were useful for pushing the analysis forward, generating ideas, and understanding individual trajectories.

The iterative process of interviewing, inductive coding, sorting, case analyses, and memo-writing gradually moved us toward a more deductive phase, in which we tentatively sketched out a model. The model, in turn, informed a new codebook of focused codes, which was used to re-code all the transcriptions and code new material (Charmaz, 2014). The model also revealed analytic gaps that were subsequently pursued through theoretical sampling of specific experiential aspects in interviews, that is, asking participants to elaborate on these as yet unanswered model elements (Charmaz, 2014). It was in this analytic phase that we included the reflection note sample. The model went through many iterations before we considered it theoretically saturated (Charmaz, 2014), that is, a model that could explain all existing and incoming data in relation to the research question on a sufficiently interesting level for it to be useful.

### Research Ethics and the Researcher’s Position

The ethical aspects of the study were formally approved by the Norwegian Center for Research Data (interview sample: Project No. 39934, reflection note sample: Project No. 52874). Prospective participants were given written information about the study, including the right to confidentiality, to anonymity, and to withdraw at any point. Not all participants returned a written consent form; however, oral informed consent was given at the beginning of the interview by all participants.

As part of researcher reflexivity, some aspects of the researchers’ background and experience may be of importance for the study. The authors’ background is from psychology, self-guided web-based programs, and counseling. Following our critical realist perspective, we acknowledge that this background necessarily has influenced what we have seen as relevant processes in the data, although we have strived for openness and inclusiveness. Another relevant aspect of our collective background is that Marianne T. S. Holter has previously worked part-time at the Norwegian Quit Smoking Line. This clinical experience with the study population was never mentioned to the participants, but probably contributed to building a rapport in the interviews.

Furthermore, Andy was designed and developed by Håvar Brendryen and Marianne T. S. Holter. This was an asset during data collection and analysis, because the self-guided web-based intervention that was the focus of the study was intimately familiar to us. However, we have been vigilant to counter any threats to the validity of the study as a cause of this role duality, both in data collection and in analysis. The validity threat related to data collection was related to the fact that Holter, who had co-developed the intervention, also conducted the interviews, and that participants may talk more positively about the intervention as a cause of that. Although we did not inform participants about this role duality, some nevertheless assumed this to be the case. In these situations, Holter made it clear that her current role was as an interviewer-researcher interested in all aspects of the participant’s experiences. The validity threat related to data analysis was that our role in development might motivate us to portray Andy in a favorable light. Feelings of pride or disappointment did indeed arise in different situations, and this is something we have regularly examined in reflexive memos. By reflexively confronting these feelings whenever we feared that they might influence the study, we believe we have focused on the participants’ perspectives and stayed true to the data. Perhaps even more importantly, the model we present does not concern how well participants liked the intervention or how “good” it is; the relational modes and patterns we present can be combined with both positive and negative program experiences. Hence, we do not believe Holter’s dual role as a program developer/interviewer has influenced the data noteworthy.

## Findings

In retrospect, our evolving understanding of the processes under study can be said to have gone through three phases. In the following, we will account for each of these phases and the inductive emergence of the two main categories: *making come-alive* and *keeping un-alive*. As all the interviews were conducted by Marianne T. S. Holter, as well as most of the analysis, some of the text is written in her voice. All texts in quotation marks are data excerpts. Excerpts are anonymized and de-identified.

### First Phase: Person or Program?

After only a few interviews, it became clear that some participants did indeed experience Andy as a person, spontaneously describing this self-guided web-based intervention “a friend” and “a therapist.” We coded these instances with “experiences Andy as a person.” Initially, this was what preoccupied us analytically. However, it was equally clear that other participants described their experiences as similar to using any other app or computer program. At first, this made me react with disappointment—after all, referring to Andy as a computer program is accurate, a factual description of the intervention. Therefore, participants who talked about Andy as a program did not seem to relate to the intervention at all, making these interviews seem without value for the present study. Despite the seemingly irrelevance of experiencing Andy as a computer program, we were reluctant to leave these interviews out of the analysis at this early stage. One reason for this was that experiencing Andy as a program seemed to be the opposite of experiencing Andy as a person, and that leaving it out of the analysis would therefore be untruthful. Another reason was that despite our initial dismay, we were also fascinated by the fact that the participants could make sense of their interaction with the program in such different ways. The intervention was highly interactive and tailored to individual use; nevertheless, it was also standardized in its way of interacting with the participants. That this same pattern of interaction could be experienced as either a person or a program caused us to consider whether experiencing Andy as a program might entail something more than just being the taken-for-granted experience. Therefore, the code “experiences Andy as a program” was introduced. Introducing this code slowly facilitated a shift in our thinking, and we increasingly started thinking about experiencing Andy as a program as an important part of the analysis.

### Second Phase: On the Trail of Complexity

The continuing analysis revealed several complexities that the simple dichotomy of person or program could not account for. Taking a step back from *relating* (which seemed to be more about experiencing Andy as a person than experiencing Andy as a program), we instead turned to analyzing ways of *making sense* of the program interaction, a mind-shift which we found to open up for complexities, which we kept coding for inductively.

One such inductive code was “getting embarrassed over one’s own personification.” When talking about Andy as a person, several participants would laugh or giggle, suggesting a degree of embarrassment over talking about an inanimate computer program as something alive. This struck us, because similar embarrassment signifiers can also be found in the existing qualitative eHealth literature. For example, Bickmore, Caruso, et al. (2005) report the following quote from a participant (who refers to the self-guided web-based intervention as “her”): “I would ask her of course and then I’d say ‘oh this is not a person [laughs]. THIS IS NOT A PERSON [laughing].’” Interestingly, Bickmore, Caruso, et al. report the laughter as if they see it as important, but they do not comment on or analyze it.

Another early code was “artificial nature revealed,” which was used to code passages where participants expressed that sometimes it became “obvious” that Andy was a computer program. On the surface, it might seem like these participants simply experienced Andy as a program. However, if it suddenly became apparent that Andy was a program, what had it been up until then? Other observations of complexity included the fact that participants who talked about Andy as a person at the same time were fully aware that Andy was a program. Furthermore, some participants seemed to have an experience of writing “to” someone else “behind the program” (i.e., the researchers or program developers) that would read what they wrote, whereas others talked as if they were interacting directly with Andy. This added to our increasing acknowledgment of the complexities involved in relating.

As our understanding evolved with increased complexity, so did the interviews, and in some interviews, participants described Andy as sort of both a program and a person. To further our understanding of this particular experience, we employed a sensitizing concept in our analysis (Charmaz, 2014): “betwixt and between,” a concept presented by Turner (1987) to describe that which is neither one thing nor another thing and at the same time is both.^1^ This seemed an accurate description of how some participants were experiencing Andy: Andy was not simply a program, nor quite a person, but rather, both program and person—and at the same time, neither. Therefore, “betwixt and between” was another early code. Participants would also sometimes find it difficult to explain exactly “what” Andy was to them or how they experienced the program. This indecisiveness would sometimes be verbalized by participants who said that it was hard to describe. Other times the uncertainty was apparent in speech that came slowly, involving much on-the-spot reflection, including unfinished words and sentences. These interview passages were marked with the code “hard to verbalize,” and seemed to, like “betwixt and between,” indicate something that breaks down established boundaries or categories and for which one therefore lacks appropriate language.

### Third Phase: Carving Out the Complexity

At this point, we knew that we were observing complexity that needed to be accounted for analytically. Furthermore, we had a growing concern that for the most part, participants who had described Andy as a person had done so unsolicited, not as a response to the questions in the interview guide. Although these unsolicited descriptions added to the data’s validity, it also worried us that there might be aspects to the users’ experiences that our interview guide was not adequately tapping into. To address these concerns, we decided to make some methodological refinements, which included changing the interview guide (as described in the “Method” section) and adding reflection notes as an additional data source. Reading through the reflection notes of the 116 participants in the reflection note pool, we theoretically sampled participants whose reflection notes explicitly or implicitly indicated a way of relating to Andy. That is, we sampled reflection notes that indicated Andy as another social actor (e.g., “I like who you are”) or referred to Andy as an un-alive thing (e.g., “I think this is a genius program!”). Reflection notes in which ways of relating were not indicated were excluded (e.g., “I think it’s an interesting way of preparing quitting smoking”).

To further our understanding of the observed complexities, we also made use of two other sensitizing concepts (Charmaz, 2014): *thinking within* and *thinking about* (Shotter, 2007). Shotter argues that thinking from *within* an activity differs from thinking *about* that activity, with consequences for how that activity is described. This led our attention to two different *relational situations*: what a participant thought *within* a program session and what she or he thought *about* Andy when not actively engaging with the program, between or after sessions.

Equipped with a refined interview guide and two new sensitizing concepts, the next interview I conducted became a turning point in the analysis. The participant consistently talked about Andy as a computer program. However, I came to the point in the interview guide where there was a hypothetical question which sounded, “If Andy had been a person, what do you think he would say about you?” The participant replied, “‘She,’ I thought it was a ‘she.’” To accommodate the participant, I rephrased the gender of Andy indicated in the question to “she,” paying no further attention to it then and there other than sensing a small sense of puzzlement. It was not until after the interview that this puzzlement took shape: why would this participant, who apparently only thought of Andy as a computer program, nevertheless think it more appropriate to call the program “she” instead of “he”? Recall, the Norwegian name of the intervention (“Endre”) is masculine (although not very common). I had chosen the name and had made all the program texts thinking that I was creating the “voice” of a male character. Thus, I knew there was nothing designed into the program to suggest that Andy was *female*. Hence, if the participant thought Andy was a “she,” there had to be some element of interpretation on her part, and I wondered whether it was possible that sometimes, or on some level, she was thinking about Andy as a person herself, although she had claimed not to. I scheduled a follow-up interview, and in this interview, I told her that although all participants know that Andy is a program, some participants nevertheless experience it as a person *within* the program sessions, upon which the participant said,

But yes, when you put it like that, I’m thinking—and you’re talking about *in that moment*—yes, there and then in that moment when I was sitting and working with the program, of course it was a person that sort of spoke to me. . . . Sort of . . . it’s that illusion that it isn’t just a program, it’s someone you’re sitting and talking to. (Female participant, interviewed)

This interview revealed that it was possible to make sense of the program interaction in one way within program sessions and in another way when thinking about the program outside of program interaction. The participant called this an “illusion,” and we made this an early code (“agrees on illusion”). This interview was a turning point also because it brought to the surface something we had sensed for some time: that participants might see the program interaction as containing elements of both program and person (even if they do not explicitly articulate this).

This re-kindled our initial perspective of relating as a process, and we coined two *relational modes* that we called *making come-alive*—thinking of Andy as a person—and *keeping un-alive*—thinking of Andy as a program (we account for these relational modes below). Our effort to turn these relational modes into relational categories caused us to make four new codes: “making come-alive about” (thinking about Andy as a person), “keeping un-alive about” (thinking about Andy as a program), “making come-alive within” (experiencing Andy as a person within program sessions), and “keeping un-alive within” (experiencing Andy as a program within program sessions). We re-coded all data material with these codes, developed the categories analytically through memos, and theoretically sampled in interviews (Charmaz, 2014). We found that these new codes could be used to explain early inductive codes that were potential relational categories: “creating a secret companion,” “accepting an illusion,” and “using a self-help tool” (Table 1).

**Table 1.** Early Relational Categories.

Relational process	Keeping un-alive outside of program sessions	Making come-alive outside of program sessions
Keeping un-alive within program sessions	Using a self-help tool	No data for this combination
Making come-alive within program sessions	Accepting an illusion	Creating a secret companion

However, we were struggling with data that did not fit neatly into these relational categories. For some participants, the names of the relational categories seemed to miss the mark (e.g., “companion” implying something more intimate or emotional than the experience the participant was conveying). What caused greater worry was that some participants’ experiences did not seem to fit within either relational category. For example, some participants talked about Andy as a program most of the time—but could suddenly in a sentence refer to Andy as “him,” or laughingly say something that portrayed Andy as a person. The participant was talking about Andy and not engaged with the program, so they were not “accepting an illusion.” Nor did these few instances of making come-alive seem to qualify as “creating a secret companion,” but it did not seem accurate to categorize such a participant as simply using a self-help tool. In sum, it was difficult to fit participants into relational categories without defying the complexity of their experiences.

In a serendipitous moment of turning the analysis inside-out, we realized that instead of trying to reduce the complexity we were observing by forcing all participants into mutually exclusive relational categories, we could retain the complexity by focusing on the relational modes making come-alive and keeping un-alive. The relational modes could still be combined with relational situations to form typical relational patterns, as we illustrated in a final iteration of the evolving model. This model could explain all existing and incoming data in terms of how a participant related to Andy, and therefore we considered it theoretically saturated. The model will be accounted for below.

### A Model of Relating in Automated Therapy

Participants related to Andy with two *relational modes: making come-alive* and *keeping un-alive*. When *keeping un-alive*, the self-guided web-based intervention was experienced as an inanimate object incapable of thinking and feeling, to which the participant had no relationship. In this relational mode, they described Andy as a “program,” a “tool,” a “machine,” a “computer,” a “robot,” a “standardized scheme,” or a “product”; somewhere they could “fill in” information, or like a “questionnaire.” Andy was a thing, not a social actor. Therefore, when keeping un-alive, participants would sometimes think of their interaction with other social actors, the people “behind” the program (the designers or the researchers), as exemplified by one participant: “I keep thinking about how you’ve sat down and made this program! There’s quite a lot of work put into this!” (female participant interviewed).

In contrast, when *making come-alive*, the self-guided web-based intervention was experienced as a social presence capable of thinking, feeling, and acting on its own accord. In this relational mode, the other social actor of the interaction was not the designers or researchers, but Andy—evidenced by participants referring to Andy as “someone” (as opposed to “something”). This “someone” “cared,” “understood,” and “supported” participants. They attributed to Andy the ability to “keep an eye” on them, have “expectations,” “have faith in me,” and “wish me well”; someone “positive,” “interested,” and “very nice”; someone who refrained from an ability to act negatively (i.e., “judge” or “pressure”). In reflection notes, making come-alive was expressed by referring to Andy as “you,” as exemplified by the following participant: “The funny thing is that I like talking to you, you give me pictures and metaphors I haven’t thought about myself” (female participant, reflection notes).

When making come-alive, it was also of significance whether participants experienced the interaction with Andy positively or negatively. There were a few examples of participants experiencing Andy negatively, leading to Andy being described as “quarreling,” “doubting,” or failing to follow up on appointments. However, the recurring experience was of Andy as a *supportive social presence*. This is of significance for the subsequent discussion of a potential person-to-program emotional bond.

The combinations of making come-alive and keeping un-alive could be described with three typical relational patterns: a *nonsocial interaction*, a *semi-social interaction*, and a *semi-social relationship*. Participants who experienced a *nonsocial interaction* kept un-alive both when thinking *about* the program and *within* their program experiences. In interviews, this was revealed through joint exploration, in which these participants expressed genuine bewilderment and incomprehension over the thought of experiencing the program sessions as conversations. Instead, they would describe interacting with Andy as “talking to myself,” “mapping” behaviors and patterns, or using a “helpful tool.” One participant said, “Then and there, when I’m answering the questions, it’s . . . maybe more like talking to myself? . . . Yes, maybe more like initiating thought process, I think would be more accurate to say” (female participant, interviewed). Reflection notes confirmed that some participants kept un-alive also when actively engaged with the program. Although they were answering Andy’s very personal question, “How would you describe working with me,” these participants would nevertheless refer to internal change processes and call Andy “the program,” as exemplified by one participant:

Like a hidden part of my personality. Nothing is new, all the thoughts about smoking and quitting smoking have been thought before, just in a different way and sequence. I get to structure my thoughts and approach free from stress. My subconsciousness is working when I’m not working with the program. (Female participant, reflection notes)

Other participants experienced a *semi-social interaction*, keeping un-alive when thinking *about* the program but making come-alive *within* program sessions. These participants would agree to “play along” then and there—but leave the alive-making once they left the session. A semi-social interaction often involved a tension between making come-alive and keeping un-alive, an ambiguousness of the program being both a social actor and not a social actor at the same time. Sometimes, participants struggled to verbalize this, but some would describe Andy explicitly as being “in the borderlands,” a “robot psychologist” or a “digital friend.” One participant explained the ambiguity this way:

It’s funny, because it becomes kind of a relationship in quotation marks . . .. What can I say . . . something in-between a program and a person, if you get what I’m saying? That it’s . . . almost neither nor. (Female participant, interviewed)

In reflection notes, ambiguity was expressed in participants’ use of quotation marks, almost as if to keep an element of un-aliveness to expressions that suggested making come-alive. This is illustrated in the following: “Nice to have a ‘friend’ who gets how hard it is to quit smoking and who helps me liberate thoughts about quitting” (male participant, reflection notes).

Finally, some participants experienced a *semi-social relationship*, in which they substantially made come-alive also when thinking about the program even when not interacting with it. In the semi-social relationship, participants seemed to experience Andy as a social presence that lingered even after the session was over. To these participants, Andy was a positive social presence held in their minds as someone who could “be there” for them or “look after” them, sometimes assigned with different (human) social roles: an “understanding friend,” a “secret friend,” a “tutor,” a “psychologist,” or a “therapist.” We call this a relationship because the experience of Andy as a supportive social presence lasted beyond the immediate interaction and seemed to involve more substance. Exemplifying this, one participant wrote the following at the end of the intervention: “It’s been nice and I’ll miss you” (female participant, reflection notes). One participant said that she could imagine what Andy looked like, indicating that she would picture “him” in her mind: “He has light hair. And he’s . . . perhaps 35. Not drop-dead gorgeous or anything, but a little thin . . . Very friendly looks. Yes. A friendly guy” (female participant, interviewed). Another participant wrote, “I completely forget that you’re a program” (female participant, reflection notes).

However, a semi-social relationship also included elements of keeping un-alive, and ambiguity was sometimes expressed through verbal modifiers (e.g., “*kind of* like a person,” “*almost* like a friend”), or by expressing embarrassment over making come-alive through laughing and giggling. Whether making come-alive or keeping un-alive, there was nothing in the participants’ accounts suggesting that their use of these relational modes was intentional or volitional.

Having established these three typical relational patterns, we returned one final time to the data material and categorized all participants according to which relational pattern she or he mostly represented. This was possible because we no longer considered them mutually exclusive categories. We classified participants conservatively; when in doubt, we chose a relational pattern implying less making come-alive. The numbers therefore likely underestimate the degree of making-come alive in the sample. Of the total 71 participants (interview sample and reflection note sample combined), two participants could not be categorized (both were early interviews conducted before the conceptualization of the relational modes with insufficient data for retrospect categorization). The remaining 69 participants could roughly be categorized as following: 12 expressed a nonsocial interaction, 34 expressed a semi-social interaction, and 23 expressed a semi-social relationship. Of course, these numbers should be interpreted with caution: because they combine two highly different data sources, because the study was not designed to estimate how these relational types are represented in the population, and because the boundary between the categories are not absolute. The reason for reporting them is to support the following claims: (a) that both making come-alive and keeping un-alive were normal relational modes for this study sample, (b) that most participants engaged in making come-alive at least some of the time, and (c) that neither relational pattern was unique to just one or two individuals.

## Discussion

**Figure 2.**
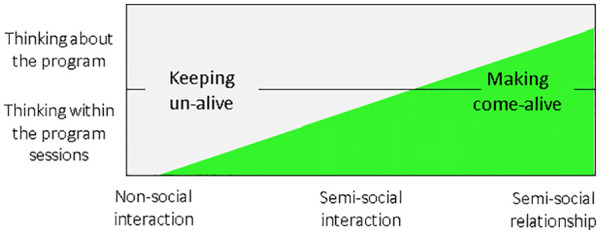
A model of relating in automated therapy, consisting of two basic relational modes: keeping un-alive (thinking about the program as an inanimate object) and making come-alive (thinking about the program as a social actor), as well as two relational situations: during program sessions and outside of program sessions. *Note.* Different combinations of the relational modes in different relational situations create three relational patterns: a nonsocial interaction (keeping un-alive all the time), a semi-social interaction (occasionally making come-alive, but keeping un-alive most of the time when not actively engaging with the program), and a semi-social relationship (alternately making come-alive and keeping un-alive when thinking about the program outside of sessions).

The purpose of this study was to develop a theoretical model of how people relate to self-guided web-based interventions and to discuss the implications of this model for a potential emotional bond of a person-to-program alliance. Regarding the emotional bond, we wanted to theoretically explain how it can be possible to experience an emotional bond to something one knows to be an inanimate computer program. In pursuing this purpose, we conducted a grounded theory study (Charmaz, 2014) with the users of a self-guided web-based intervention for quitting smoking (“Endre,” or “Andy”). Sixteen program users were interviewed and additionally 55 participated through written answers within the program (i.e., “reflection notes”). The analysis is summarized in a model of relating in automated therapy (Figure 2), in which relating is described as consisting of two relational modes: *making come-alive* (talking and thinking about the program as a social actor) and *keeping un-alive* (talking and thinking about the program as an inanimate object). Using these relational modes to a varying degree, participants exemplified different relational patterns, forming a landscape ranging from a *nonsocial interaction* (only keeping un-alive), to a *semi-social interaction* (making come-alive when interacting with the program but keeping un-alive outside of program interaction), to a *semi-social relationship* (making come-alive also after and between program sessions).

When making come-alive, participants experienced Andy as a person, confirming previous findings of how people can sometimes think of self-guided web-based interventions as human beings (Bickmore Caruso, et al., 2005; Bickmore, Gruber, & Picard, 2005; Bickmore & Picard, 2005; Brandt et al., 2013; Kaplan et al., 2003). The proposed model can place the findings of these former studies within a relational landscape. What Kaplan et al. (2003) called “feelings of guilt” (feeling judged or lectured by the program) could be described as making come-alive but experiencing the social presence negatively. Furthermore, Bickmore, Caruso, et al. (2005) report that some participants described the intervention as “somewhere between a computer and a person” (p. 730) and Kaplan et al. (2003) found that participants expressed “ambiguity and ambivalence.” This can be described as a semi-social interaction and explained as the tension between making come-alive and keeping un-alive. These two studies also provide data excerpts of participants laughing when talking about the program as a person (Bickmore, Caruso, et al., 2005; Kaplan et al., 2003). We document this tendency as well and explain it as nonverbal expressions of the embarrassment which for some is associated with the tension between making come-alive and keeping un-alive. Finally, the many examples of participants experiencing positive social emotions toward self-guided web-based interventions (Bickmore, Caruso, et al., 2005; Bickmore, Gruber, & Picard, 2005; Bickmore & Picard, 2005; Brandt et al., 2013; Kaplan et al., 2003) are explained by the proposed model as a semi-social relationship and an experienced supportive social presence.

However, does the experienced supportive social presence of a semi-social relationship amount to a person-to-program emotional bond? The emotional bond of the alliance was previously defined as involving complex, positive social emotions that assume another social actor who can think and feel. Participants with a semi-social relationship to Andy experienced Andy as such a supportive social presence, attributing to Andy the ability to feel (“care”), think (“understand,” “wish me well”), and act (refrain from judging, “keep an eye on”). It seems reasonable to call this a type of emotional bond, enabled by making come-alive and experiencing the interaction positively. Thus, 23 of the 69 participants in the current study expressed a type of emotional bond to Andy.

Why so many participants experienced an emotional bond might be explained if we consider making come-alive as a form of “transitional experience” (Winnicott, 1969). Transitional experiences are when a person creates a fantasy; an internal experience of something that is not, strictly speaking, “real,” but part of the person’s imagination brought into the world. The person temporarily acts as if this product of his or her imagination is real, and “the boundaries between what is real and what is not real is temporarily blurred” (Safran & Muran, 2003). The term “transitional experience” was launched by Winnicott (1969), who demonstrated it as a normal part of a child’s development and life-world. Transitional experiences can also be used as a tool in adult psychotherapy, as a way of acting out a scene with someone who is not really there (Safran & Muran, 2003). Considering making come-alive as a transitional experience can explain what may seem a paradox: That although participants *knew* they were interacting with an inanimate computer program, they nevertheless experienced Andy as a supportive social presence—temporarily blurring out the boundaries between real and not real in a form of “play” or “illusion.” Considering making come-alive—and the emotional bond it can facilitate—from this perspective makes it less mysterious and controversial. It becomes possible to consider an emotional bond as a potentially common way of relating to a self-guided web-based intervention.

This proposition is supported by the other reports of what we may now define as a type of emotional bond to three other self-guided web-based interventions: “Laura” (Bickmore, Caruso, et al., 2005; Bickmore, Gruber, & Picard, 2005; Bickmore & Picard, 2005), “Dit Digitale Stopprogram” (DDS, translates “Your Digital Quit Program”; Brandt et al., 2013), and Telephone-Linked Care (TLC; Kaplan et al., 2003). Similar to Andy, both Laura and TLC are interventions with relational agents; Laura is an animated figure that also communicates with body language and facial expressions (Bickmore & Picard, 2005), whereas TLC was telephone-based involving a recorded actor’s voice (Kaplan et al., 2003). This makes it tempting to conclude that a person-to-program emotional bond depends on an intervention that involves a relational agent that imitates social behavior. However, DDS looks like a traditional web-page with a side-bar menu (Brandt et al., 2013), suggesting that a person-to-program emotional bond is not entirely dependent on a relational agent. This seems to echo Nass and Reeves’s (1996) findings of participants behaving socially even toward very simple technology. This suggests a possible area for future research: how design features influence making come-alive (and keeping un-alive), and whether there are certain features that are necessary to facilitate making come-alive.

This is also a question of the proposed model’s transferability (Guba, 1981) to other contexts, that is, other self-guided web-based interventions. Another question that can be raised about the model’s transferability is whether there are cultural aspects that will influence how people relate to self-guided web-based interventions. The current study was conducted in Norway. We also suggest that the model can explain findings from studies that have been conducted in the United States (Bickmore, Caruso, et al., 2005; Bickmore, Gruber, & Picard, 2005; Kaplan et al., 2003) and Denmark (Brandt et al., 2013), suggesting that the model may at least be transferable to these other cultural contexts. Ultimately, the question of transferability to other cultures is an empirical question.

We have so far focused the discussion on one element of the proposed model, namely, the semi-social relationship, which confirms and extends on existing research. However, this study also documents the possibility of experiencing a semi-social *interaction*, which may be a useful concept for describing a hitherto unnoticed group of program users. While many users of self-guided web-based interventions will talk about these interventions as inanimate computer programs, the proposed model suggests that a proportion of these users may nevertheless make come-alive within the immediate program interaction. Given a positive program experience, these users may in the moments of using the program experience it as a supportive social presence, able to understand, care for, and support them. However, whether these passing experiences of a supportive social presence can be described as an emotional bond is a question that we are hesitant to answer. Another possible area for future research is therefore whether a person-to-program emotional bond is dependent on the experience of the program as a supportive social presence also after and between program sessions, or whether perhaps these are two different types of emotional bonds.

Another contribution this study makes is by conceptualizing keeping un-alive as a way of relating. Kaplan et al. (2003) report that some participants explicitly talked about the intervention as a machine; however, they treat this as part of their “ambiguity and ambivalence”-category and have not analyzed it as a separate relational tendency. Perhaps because thinking about programs as inanimate objects is the expected state of affairs, it has previously not been thought about as a way of relating as such. However, even though keeping un-alive might be the expected state of affairs, it is nevertheless of analytic interest; attending to keeping un-alive makes it possible to explain more variation in ways of relating, painting a fuller picture of how people relate to self-guided web-based interventions than by focusing on processes like making come-alive alone.

We have suggested ways in which the proposed model can be used to explain the experiences of people who use self-guided web-based health interventions. However, to achieve such a feat, the model must first of all be a valid representation of the experiences of the current study’s participants. Given our critical realist perspective (Maxwell, 2013), we believe that such a true representation is possible, but dependent on efforts to reduce the unintended influence of our subjective experiences and other potential threats to validity. Efforts to increase the study’s validity include grounded theory’s commitment to staying close to the data and the method of constant comparison (Charmaz, 2014; Creswell, 2013), the adding of reflection notes for method-ological triangulation (Maxwell, 2013), in situ interview checks of the researcher-interviewer’s interpretations (Brinkmann, 2007), a rigorous analysis using a variety of methods, reflexivity, and memo-writing throughout the study (Finlay, 2002, 2012).

However, the study has certain limitations. Three interviews were not audiotaped, causing loss of detailed data for these participants. Another limitation is related to the study being based on a convenience sample (as opposed to a more strategic sample), which was one reason why the model cannot explain whether different relational patterns were related to participant characteristics such as gender, age, or how long they used the intervention. Furthermore, although we suggest that experiencing a person-to-program emotional bond is likely to be a common way of relating to self-guided web-based intervention, normality cannot be ascertained with this type of study design, as it would require a larger-scale quantitative study. Nor does this model include possible consequences of different ways of relating in terms of their clinical significance or effects on program use and engagement. These questions were not possible to pursue in the current article due to the scope of accounting for and developing the presented model of relating. In a separate publication (Holter, Ness, Johansen, & Brendryen, 2019), we present an analysis of how one specific change-process, which we call “getting change-space,” was influenced by making come-alive and keeping un-alive.

The present study also has several strengths, including the methodological refinement process we undertook, as well as the previously accounted for efforts to increase validity. Following the recommendations of Levitt et al. (2017), the data are contextualized and rich and the findings are coherent. Furthermore, we performed a rigorous analysis involving many analytic tools and steps until we reached a theoretically saturated model, which is based on the experiences of a heterogeneous sample.

One practical implication of the proposed model is that to facilitate an emotional bond, an intervention should support making come-alive, but also be experienced positively and as supporting. Another area in which the model may have practical implications is in how the emotional bond of a potential person-to-program alliance is measured: The model suggests that an emotional bond is possible, but dependent on making come-alive, which alternates with keeping un-alive. Thus, whether a person filling out an alliance-measure rates the program as a supportive social presence (e.g., “understanding,” “respecting”) depends on whether she or he is making come-alive in that specific moment.

It is our belief that the proposed model of relating in automated therapy can be useful for addressing currently unanswered questions of how self-guided web-based health interventions work. The proposed model suggests that experiencing an emotional bond may be one common way of relating to self-guided web-based interventions, but that there also are other ways of relating that call for further research. The current study also raises other questions concerning how different relational modes and patterns arise (related to characteristics with the program user as well as the intervention) and what their consequences are. However, we believe the study’s main contribution lies in proposing a set of concepts for describing how people relate to self-guided web-based interventions, which may be used as a stepping stone for further research into potential associations between relating and change. These efforts may be further facilitated through the theoretical explanation provided of the person-to-program emotional bond, which may be tested and elaborated, and hopefully lead to an increased understanding of whether a working alliance plays a role in the change facilitated by automated therapy.

## Notes

1. Thank you to Professor Anne-Lise Middelthon at the Faculty of Medicine of the University of Oslo, for pointing to the relevance of “betwixt and between” for our study.
